# Recovery of an injured corticoreticular pathway via transcallosal fibers in a patient with intracerebral hemorrhage

**DOI:** 10.1186/1471-2377-14-108

**Published:** 2014-05-19

**Authors:** Sung Ho Jang, Sang Seok Yeo

**Affiliations:** 1 Department of Physical Medicine and Rehabilitation, College of Medicine, Yeungnam University 317–1, Daemyungdong, Namku, Taegu 705-717, Republic of Korea; 2 Department of Physical Therapy, College of Health Sciences, Dankook University, 119, Dandae-ro, Dongnam-gu, Cheonan-si, Chungnam 330-714, Republic of Korea

**Keywords:** Corticoreticular pathway, Corticoreticulospinal tract, Transcallosal fibers, Diffusion tensor tractography, Stroke

## Abstract

**Background:**

Several studies have reported on injury of the corticoreticular pathway (CRP), however, little is known about the mechanism for recovery of an injured CRP. We report on a patient with intracerebral hemorrhage (ICH) who showed recovery of an injured CRP via transcallosal fibers, which was demonstrated by diffusion tensor tractography (DTT).

**Case presentation:**

A 67-year-old man presented with complete paralysis (Medical Research Council: 0/5) of the left extremities at the onset of a right putaminal hemorrhage. At six weeks after onset, he presented with more severe weakness of proximal joint muscles than distal joint muscles (right shoulder abductor; 2^−^, finger extensor; 3^+^, hip flexor; 2^+^, ankle dorsiflexor; 3). Although his right hemiplegia had recovered well, he consistently showed more severe proximal weakness (right shoulder abductor; 3, finger extensor; 4, hip flexor; 3^+^, ankle dorsiflexor; 4) until 16 weeks after onset. On both six- and 16-week DTTs, in the left (affected) hemisphere, the CRP showed severe narrowing with discontinuation of the anterior fibers at the corona radiata on six-week DTT, however, the discontinued anterior fibers of the CRP were connected to the right cerebral cortex via transcallosal fibers on 16-week DTT.

**Conclusion:**

We demonstrated recovery of an injured CRP via transcallosal fibers in a patient with ICH. We believe that this might be a mechanism for recovery of an injured CRP.

## Background

The corticoreticulospinal tract, one of the extrapyramidal motor pathways, consists of the corticoreticular pathway (CRP) and the reticulospinal tract. This tract innervates proximal muscles of the extremities and axial muscles [1-3]. Exact evaluation of the CRP had been impossible in the live human brain. However, Diffusion tensor tractography (DTT), which is derived from diffusion tensor imaging (DTI), has enabled three-dimensional visualization and estimation of the CRP [4]. Several studies have reported on injury of the CRP in various brain pathologies, including intracerebral hemorrhage (ICH), cerebral infarct, and traumatic brain injury [5-7]. However, knowledge regarding the mechanism for recovery of an injured CRP is limited [8].

In this study, we report on a patient with ICH who showed recovery of an injured CRP via transcallosal fibers, which was demonstrated by DTT.

## Case presentation

**Figure 1:**
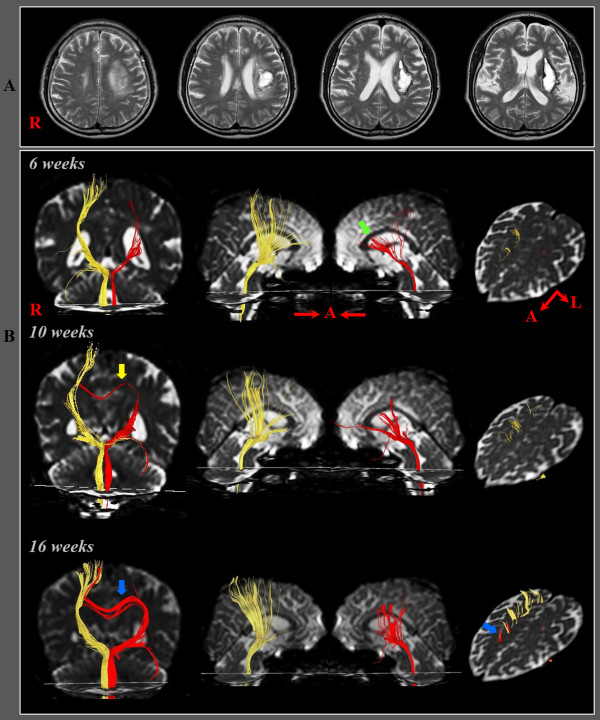
**(A) T2-weighted MR images showed a hematoma in the left corona radiata and basal ganglia at six weeks after onset (B) Results of diffusion tensor tractography (DTT).** On six-, 10- and 16-week DTTs, the CRPs in the right hemispheres originated from the premotor cortex and primary motor cortex, and descended through the known CRP pathway. By contrast, in the left (affected) hemisphere, the CRP showed severe narrowing with discontinuation of the anterior fibers (green arrow) on six-week DTT, however, the discontinued fibers of the CRP were connected to the corpus callosum through the transcallosal fibers (yellow arrow) on 10-week DTT, and became thicker and connected to the right cerebral cortex via the corpus calllosum (blue arrow) on 16-week DTT.

A 67-year-old right-handed man presented with complete paralysis of the right upper and lower extremities (Medical Research Council [MRC]: 0/5) at the onset of a spontaneous ICH (Table 1). At six weeks after ICH onset, he was admitted to the rehabilitation department of a university hospital after undergoing rehabilitation for three weeks at the previous university hospital. Brain MR images showed a hematoma in the left corona radiata and basal ganglia at six weeks after onset (Figure 1-A). He presented with severe weakness of the right upper and lower extremities with more severe weakness of proximal joint muscles than distal joint muscles (MRC: right shoulder abductor; 2^−^, finger extensors; 3^+^, hip flexor; 2^+^, ankle dorsiflexor; 3) (Table 1). He was administered comprehensive rehabilitative therapy, which included neurotropic drugs (dopaminergic drug [pramipexole, amantadine, and levodopa] and antidepressant [venlafaxine]), movement therapy, and neuromuscular electrical stimulation of the affected shoulder abductor, finger extensor, and ankle dorsiflexor [9,10]. Movement therapy was conducted with a focus on improvement of right upper and lower extremity functions, and was conducted five times per week in our physical and occupational therapy department. Although the weakness of his right side recovered well for 10 weeks until 16 weeks after onset, he consistently showed more severe proximal weakness (right shoulder abductor; 3, finger extensor; 4, hip flexor; 3^+^, ankle dorsiflexor; 4). As a result, he was able to perform some fine motor activities using the right hand and to walk independently at 16 weeks after onset. The patient provided informed consent, and the study protocol was approved by our institutional review board.

**Table 1:** Longitudinal changes in motor function

**Duration from onset**		**Onset**	**6 weeks**	**10 weeks**	**16 weeks**
MRC	Shoulder abductor	0	2^−^	2	3
	Elbow flexor	0	3	3^+^	3^+^
	Finger flexor	0	3^+^	3^+^	4
	Finger extensor	0	3^+^	3^+^	4
	Hip flexor	0	2^+^	3	3^+^
	Knee extensor	0	3^−^	3^+^	4
	Ankle dorsiflexor	0	3	4	4
MEP	Latency		21.7	22.6	21.8
	Amplitude		300	800	2400
	ET(%)		95	95	80

MRC: Medical Research Council scale. 0; no contraction, 1; palpable contraction, but no visible movement, 2; movement without gravity, 3; movement against gravity, 4; movement against a resistance lower than the resistance overcome by the healthy side, 5; movement against a resistance equal to the maximum resistance overcome by the healthy side. MEP: motor evoked potential, ET: excitatory threshold.

### Diffusion tensor tractography

DTI scanning was performed at six, 10 and 16 weeks after onset, using a multi-channel head coil on a 1.5-T Philips Gyroscan Intera (Philips, Best, Netherlands) with single-shot echo-planar imaging. For each of the 32 noncollinear diffusion-sensitizing gradients, we acquired 67 contiguous slices (matrix = 192 x 192, FOV = 240 mm, TR/TE = 10,726/76 ms, b = 1000 mm^2^s^−1^, NEX = 1, thickness = 2.5 mm). Three-dimensional reconstructions of fiber tracts were obtained using the DTI task card software (Philips Extended MR Work Space 2.6.3) (threshold fractional anisotropy = 0.15, angle = 27°) [11]. The CRP was determined by selection of fibers passing through the seed and target regions of interest (ROI). A seed ROI was placed on the reticular formation of the medulla. The target ROI was placed on the midbrain tegmentum [1,4,7].

On six-, 10- and 16-week DTTs, the CRPs in the right hemispheres originated from the premotor cortex and primary motor cortex, and descended through the known CRP pathway. By contrast, in the left (affected) hemisphere, the CRP showed severe narrowing with discontinuation of the fibers at the corona radiata on six-week DTT. On the other hand, the discontinued anterior fibers of the CRP were connected to the corpus callosum through the transcallosal fibers on 10-week DTT, and became thicker and connected to the right cerebral cortex via the corpus calllosum on 16-week DTT (Figure 1-B).

### Transcranial magnetic stimulation

Transcranial magnetic stimulation (TMS) was performed to demonstrate the recovery process of the corticospinal tract using a Magstim Novametrix 200 magnetic stimulator with a 9-cm mean diameter circular coil (Novametrix Inc). Cortical stimulation was performed with the coil held tangentially over the vertex. The left hemisphere was stimulated by a counterclockwise current and the right hemisphere was stimulated by a clockwise current. Motor-evoked potentials (MEPs) were obtained from both abductor pollicis brevis muscles in a relaxed state. The excitatory threshold (ET) was defined as the minimum stimulus required to elicit an MEP with a peak-to-peak amplitude of 50 μV or greater, in two out of four attempts. Stimulation intensity was set at the ET plus 20% of the maximum stimulator output. Each site was stimulated three times at intervals with a minimum of 10 seconds, from which the shortest latency and the average peak to peak amplitudes were adopted.

On the six-week TMS study, an MEP with low amplitude was evoked (latency: 21.7 msec, amplitude: 0.3 mV ET: 95%), and on the 10-week and 16-week TMS study, the amplitude was gradually increased (10 weeks - latency: 22.8 msec, amplitude: 0.8 mV, ET: 95%; 16 weeks - latency: 21.8 msec, amplitude: 2.4 mV, ET: 80%).

## Discussion

In the current study, we attempted to demonstrate the recovery of an injured CRP by following up on the change of the injured CRP from six weeks to 10 weeks and 16 weeks during a 10-week rehabilitation period. The left injured CRP showed discontinuation in the anterior fibers at the corona radiata level with preservation of the smaller posterior portion on six-week DTT and the anterior discontinued fibers were connected to the corpus callosum through the transcallosal fibers on 10-week DTT, and became thicker and connected to the right (unaffected) cortex via transcallosal fibers on 16-week DTT. During the 10-week rehabilitation period, the patient showed good motor recovery in both proximal and distal joint muscles (MRC: right shoulder abductor; 2^−^ - > 3, finger extensor; 3^+^ - > 4, hip flexor; 2^+^ - > 3^+^, ankle dorsiflexor; 3 - > 4). The motor recovery of distal joint muscles appears to be compatible with the increase of amplitude of MEP in the right hand muscle, which indicates an increase in the number of CST fibers [12,13]. The recovery of proximal joint muscles appears to be mainly attributed to the change of the left CRP: the anterior torn fibers of the left CRP at the corona radiata level were connected to the right (unaffected) cerebral cortex via transcallosal fibers. Therefore, we believe that this change of the injured CRP via transcallosal fibers might be a mechanism for recovery of an injured CRP.

To the best of our knowledge, recovery of an injured CRP has only been reported in one patient, using DTT [8]. In 2013, Yeo and Jang reported on a patient who presented with recovery of a discontinuation of the left CRP at the midbrain level at three weeks after recovery of an ICH through the normally existing pathway of the CRP to the left cerebral cortex at six weeks [8]. In their patient, the motor outcome in the proximal joint muscles (right shoulder flexor; 4, hip flexor; 4) at six weeks after onset was better than the motor outcome of our patient (the right shoulder abductor; 3, hip flexor; 3^+^) at 16 weeks after onset. This finding suggests that recovery via transcallosal fibers might accompany poorer motor outcome than recovery via the normally existing neural pathway of the CRP. On the other hand, with regard to the CST, a few studies have reported on motor recovery by transcallosal fibers in stroke patients [14-16]. Consequently, to the best of our knowledge, our study is the first study to demonstrate motor recovery by transcallosal fibers of the CRP in a stroke patient.

## Conclusion

We demonstrated recovery of an injured CRP via transcallosal fibers in a patient with ICH. We believe that this might be one of the mechanisms for recovery of an injured CRP. In addition, when comparing motor outcome with that of previous study [8], our results suggest that recovery via transcallosal fibers appeared to be associated with poorer motor outcome than recovery via the normally existing neural pathway of the CRP. However, this study is limited because it is a single case study. Conduct of further studies including larger case numbers is warranted. In addition, studies on rehabilitation strategies to induce or facilitate this recovery mechanism should be encouraged. Further studies on the other recovery mechanism following a CRP injury are also needed.

## Consent

Written informed consent was obtained from the patient for publication of this case report and accompanying images. A copy of the written consent is available for review by the Editor-in-Chief of this journal.

## Abbreviations

CRP: Corticoreticular pathway; DTI: Diffusion tensor imaging; DTT: Diffusion tensor tractography; ICH: Intracerebral hemorrhage; MRC: Medical research council; ROI: Regions of interest; TMS: Transcranial magnetic stimulation; MEPs: Motor-evoked potentials; ET: Excitatory threshold.

## Competing interests

The authors declare that they have no competing interests.

## Authors’ contributions

SHJ: conceiving and designing the study, funding, data acquisition, manuscript development and manuscript writing. SSY: manuscript development, data acquisition, manuscript writing and manuscript authorization. Both authors read and approved the final manuscript.

## Pre-publication history

The pre-publication history for this paper can be accessed here:

http://www.biomedcentral.com/1471-2377/14/108/prepub

## References

[B1] Matsuyama K Mori F Nakajima K Drew T Aoki M Mori S Locomotor role of the corticoreticular-reticulospinal-spinal interneuronal system Prog Brain Res 2004 143 239 249 14653169 10.1016/S0079-6123(03)43024-0

[B2] Mendoza JE Foundas AL Clinical Neuroanatomy: A Neurobehavioral Approach 2007 NewYork /London: Springer

[B3] Miyai I Yagura H Oda I Konishi I Eda H Suzuki T Kubota K Premotor cortex is involved in restoration of gait in stroke Ann Neurol 2002 52 2 188 194 10.1002/ana.10274 12210789

[B4] Yeo SS Chang MC Kwon YH Jung YJ Jang SH Corticoreticular pathway in the human brain: diffusion tensor tractography study Neurosci Lett 2012 508 1 9 12 10.1016/j.neulet.2011.11.030 22197953

[B5] Do KH Yeo SS Lee J Jang SH Injury of the corticoreticular pathway in patients with proximal weakness following cerebral infarct: diffusion tensor tractography study Neurosci Lett 2013 546 21 25 23643994 10.1016/j.neulet.2013.04.040

[B6] Kwon HG Jang SH Delayed gait disturbance due to injury of the corticoreticular pathway in a patient with mild traumatic brain injury Brain Inj 2014 In press 10.3109/02699052.2014.887228 24564187

[B7] Jang SH Chang CH Lee J Kim CS Seo JP Yeo SS Functional role of the corticoreticular pathway in chronic stroke patients Stroke 2013 44 4 1099 1104 10.1161/STROKEAHA.111.000269 23444306

[B8] Yeo SS Jang SH Recovery of an injured corticospinal tract and an injured corticoreticular pathway in a patient with intracerebral hemorrhage NeuroRehabilitation 2013 32 2 305 309 23535792 10.3233/NRE-130848

[B9] Kwon HG Jang SH Significance of rehabilitative management during the critical period for motor recovery in intracerebral hemorrhage: a case report J Rehabil Med 2012 44 3 280 284 10.2340/16501977-0931 22367566

[B10] Scheidtmann K Fries W Muller F Koenig E Effect of levodopa in combination with physiotherapy on functional motor recovery after stroke: a prospective, randomised, double-blind study Lancet 2001 358 9284 787 790 10.1016/S0140-6736(01)05966-9 11564483

[B11] Kunimatsu A Aoki S Masutani Y Abe O Hayashi N Mori H Masumoto T Ohtomo K The optimal trackability threshold of fractional anisotropy for diffusion tensor tractography of the corticospinal tract Magn Reson Med Sci 2004 3 1 11 17 10.2463/mrms.3.11 16093615

[B12] Jang SH Cho SH Kim YH You SH Kim SH Kim O Yang DS Son S Motor recovery mechanism of diffuse axonal injury: a combined study of transcranial magnetic stimulation and functional MRI Restor Neurol Neurosci 2005 23 1 51 56 15846032

[B13] Rossini PM Barker AT Berardelli A Caramia MD Caruso G Cracco RQ Dimitrijevic MR Hallett M Katayama Y Lucking CH Maertens de Noordhout AL Marsden CD Murray NMF Rothwell JC Swash M Non-invasive electrical and magnetic stimulation of the brain, spinal cord and roots: basic principles and procedures for routine clinical application. Report of an IFCN committee Electroencephalogr Clin Neurophysiol 1994 91 2 79 92 10.1016/0013-4694(94)90029-9 7519144

[B14] Chang MC Jung YJ Jang SH Motor recovery via transcallosal and transpontine fibers in a patient with intracerebral hemorrhage Am J Phys Med Rehabil 2014 In press 10.1097/PHM.0000000000000076 24658429

[B15] Jang SH Park KA Ahn SH Cho YW Byun WM Son SM Choi JH Kwon YH Transcallosal fibers from corticospinal tract in patients with cerebral infarct NeuroRehabilitation 2009 24 2 159 164 19339754 10.3233/NRE-2009-0464

[B16] Jang SH Transcallosal motor pathway from affected motor cortex to affected hand in a patient with corona radiate infarct: a diffusion tensor tractography and transcranial magnetic stimulation stud Neural Regen Res 2010 5 1117 1120

